# Predicting femoral neck screw pullout strength using DXA and novel assessments of bone microarchitecture

**DOI:** 10.1007/s11657-026-01763-0

**Published:** 2026-08-21

**Authors:** Jarod Moyer, Richard Behlmer, Diane Krueger, Gretta Borchardt, Neil Binkley, Herman Feller, Sam Mosiman, Paul Whiting, Josh Roth

**Affiliations:** 1 Department of Orthopedics & Rehabilitation, University of Wisconsin School of Medicine and Public Health, 1685 Highland Avenue, 6th Floor, Madison, WI 53705 USA; 2 Osteoporosis Clinical Research Program, 2870 University Ave. Ste. 100, Madison, WI 53705 USA

**Keywords:** Quantitative computed tomography, Two-dimensional dual-energy x-ray absorptiometry, Trabecular bone score, 3D-Shaper

## Abstract

***Summary*:**

This study determined how well DXA and other novel assessments of bone microarchitecture predicted screw pullout strength in the femoral neck. Hip-specific DXA performed as well as the novel approaches at predicting screw pullout. These findings support the use of DXA to evaluate bone quality and aid in surgical decision-making.

**Purpose:**

The primary purpose of our study was to determine how well dual-energy x-ray absorptiometry (DXA), trabecular bone score (TBS Ortho), 3D-Shaper, and quantitative computed tomography (qCT) predicted screw pullout of cannulated screws placed in the femoral neck of cadaveric specimens. Our secondary objective was to determine how well insertional torque predicted screw pullout.

**Methods:**

We conducted a biomechanical study using twenty proximal cadaveric femurs. Each femur underwent imaging with each respective modality to assess bone quality. Screws were placed in a standardized configuration, and insertional torque was measured using a digital torque screwdriver. Screws were then pulled out using a mechanical testing machine to determine peak pullout strength. The relationship between screw pullout force and each bone metric was assessed using Spearman correlation coefficients and univariate linear mixed-effects models.

**Results:**

DXA bone mineral density (BMD) and 3D-Shaper volumetric BMD (vBMD) at the femoral neck had the highest correlation coefficients with screw pullout strength (*r* = 0.95, *p* < 0.001). However, all of the imaging modalities were found to be strong predictors of screw pullout. Insertional torque was also a strong predictor of screw pullout strength (*r* = .86, *p* < 0.001).

**Conclusion:**

Our findings highlight the clinical utility of hip-specific DXA as a strong and accessible predictor of screw pullout strength, performing as well as newer imaging modalities such as TBS Ortho and 3D-Shaper. Insertional torque also demonstrated strong predictive value and may serve as a useful intraoperative tool when preoperative bone density data are not available.

**Supplementary Information:**

The online version contains supplementary material available at 10.1007/s11657-026-01763-0.

## Introduction

Femoral neck fractures are common injuries, seen primarily in geriatric patients as a result of low-energy falls [1]. These fractures carry substantial morbidity and mortality, with 1-year mortality rates as high as 33% [2]. Operative treatment for these injuries involves either surgical fixation or arthroplasty. In current practice, the decision between these options is based primarily on the fracture pattern and the degree of fracture displacement. Displaced fractures are commonly treated with arthroplasty—typically hemiarthroplasty—whereas non-displaced and valgus impacted fracture patterns are typically treated with surgical fixation in the form of cannulated screws or a fixed-angle device. However, multiple complications may occur after femoral neck fracture fixation in geriatric patients, including screw cut-out, fixation failure, and fracture shortening [3]. Consequently, some surgeons have begun to question whether fracture characteristics alone should determine the recommended surgery or if bone quality should also be considered [4, 5].

Several medical imaging modalities are available to evaluate bone quality in vivo. The current clinical standard for the diagnosis of osteoporosis is bone mineral density (BMD), as determined by either two-dimensional dual-energy x-ray absorptiometry (DXA) or quantitative computed tomography (qCT) [6, 7]. BMD, using both of these imaging modalities, correlates with fracture risk as well as screw pullout strength [8–10]. However, BMD alone does not adequately predict fracture risk, as the majority of “osteoporosis-related” fractures occur in patients who would be determined to have non-osteoporosis bone based on BMD criteria [11]. Trabecular bone score (TBS) is a metric that can be extracted from a DXA and was designed to provide a more refined assessment of bone microarchitecture [12]. Clinically, TBS is applied to the lumbar spine; however, the Texture Research Investigational Platform (TRIP) software (Medimaps, Geneva, Switzerland) has adapted this approach to generate a bone texture score for non-spine anatomy referred to as TBS Ortho [12]. In biomechanical studies, TBS correlated with vertebral body stiffness, and TBS has recently been shown to predict hip fracture risk independent of BMD [13–17]. Similarly, 3D-Shaper (Galgo Medical, Barcelona, Spain) provides a patient-specific 3D CT-like analysis from a standard 2D DXA image. This has been shown to improve hip fracture risk prediction over conventional BMD obtained via DXA [14]. In addition to these various imaging modalities, applied insertional torque has been shown to be correlated with bone quality and may be measured intraoperatively [10, 18–20].

Recently, a study by Bernatz et al. found that TBS Ortho correlates more strongly than DXA and qCT to screw pullout strength in a distal femur cadaveric model [21]. Screw pullout is a routinely used parameter to quantify screw-bone interface strength [22, 23]; however, that study did not evaluate 3D-Shaper, and the results may not be generalizable to the proximal femur due to known differences in cortical and trabecular bone composition and arrangement. To our knowledge, no study has investigated screw pullout strength in the femoral neck using these newer microarchitecture analyses. The primary objective of our study, therefore, was to determine how well DXA, TBS Ortho, 3D-Shaper, and qCT predicted screw pullout of cannulated screws placed in the femoral neck of cadaveric specimens. We hypothesized that TBS Ortho, which has been used as a surrogate for bone microarchitecture, would have a strong correlation with screw pullout strength. Our secondary objective was to determine how well insertional torque predicted screw pullout.

## Methods

This was a laboratory-based biomechanical study using cadaveric femurs to evaluate the relationship between bone quality metrics and screw pullout strength. Imaging, screw placement, and mechanical testing were all performed at a single academic institution. We procured ten paired proximal cadaver femurs, for a total of 20 femurs (i.e., left and right femurs from the same cadaver), stripped of soft tissue. Donors ranged in age from 56 to 96 years, with one male and one female represented in each decade. Specimens had no known history of conditions that would alter bone mineral density (e.g., osteoporosis, osteomalacia, metabolic bone disease, skeletal malignancy, prior fracture, or previous surgery). The femurs were stored in −4 °C freezers until testing was performed, at which point they were thawed and kept hydrated in the refrigerator until ready for testing. The specimens were then unwrapped, had the screws placed, and underwent mechanical testing. This entire process lasted approximately 20 min per specimen.

### Imaging

**Fig. 1:**
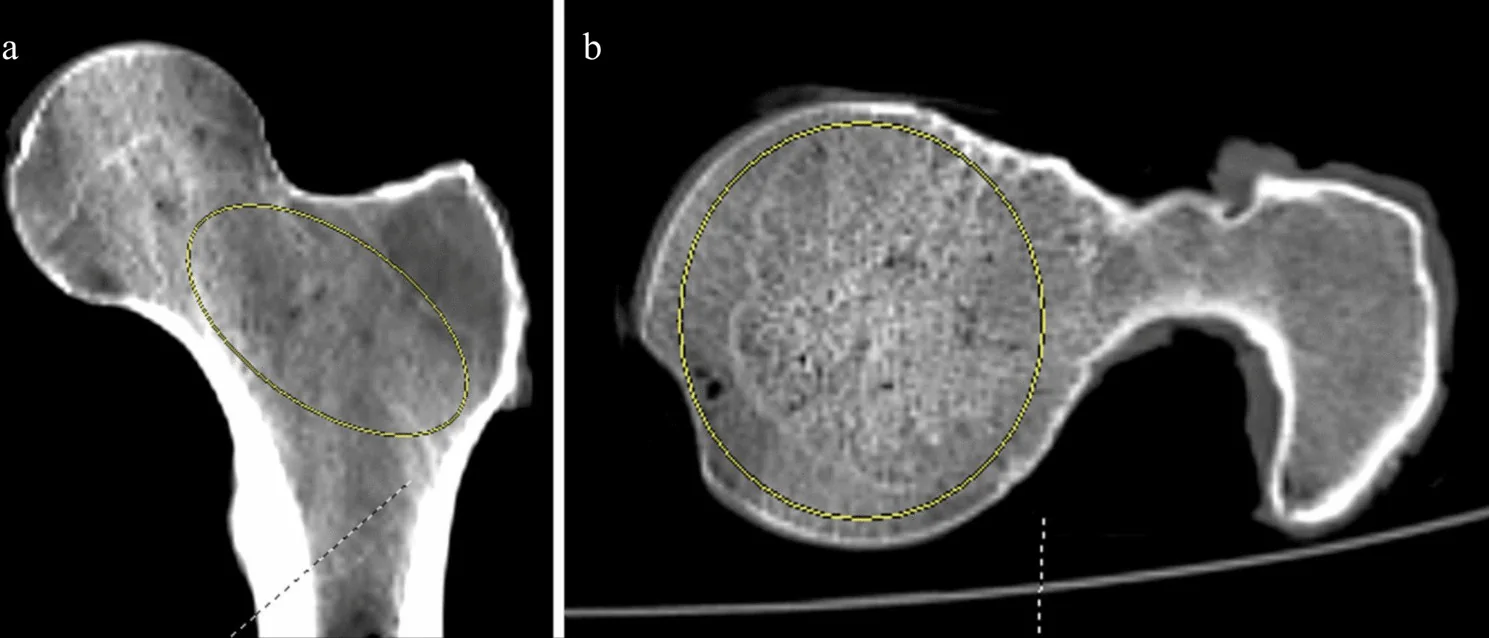
**a** Coronal and **b** axial qCT images showing elliptical regions of interest used to calculate Hounsfield units for the femoral neck and femoral head, respectively

Each femur underwent multiple imaging modalities to assess bone quality. CT scans were obtained using a Discovery CT750 HD scanner (General Electric, Madison, WI). We used Picture Archiving and Communication Systems (PACS) for measurements. CT Hounsfield units were measured using an elliptical region of interest (ROI) in the peritrochanteric region of coronal CT slices which we referred to as qCT femoral neck (Fig. 1a). This location was chosen to mimic the ROI for standard DXA imaging of the hip. We also took measurements in axial slices of the femoral head which we referred to as qCT femoral head (Fig. 1b). Measurements were performed 3 times by one author (RJB) and averaged. This ROI was drawn as large as possible without including the cortical bone (Fig. 1).

**Fig. 2:**
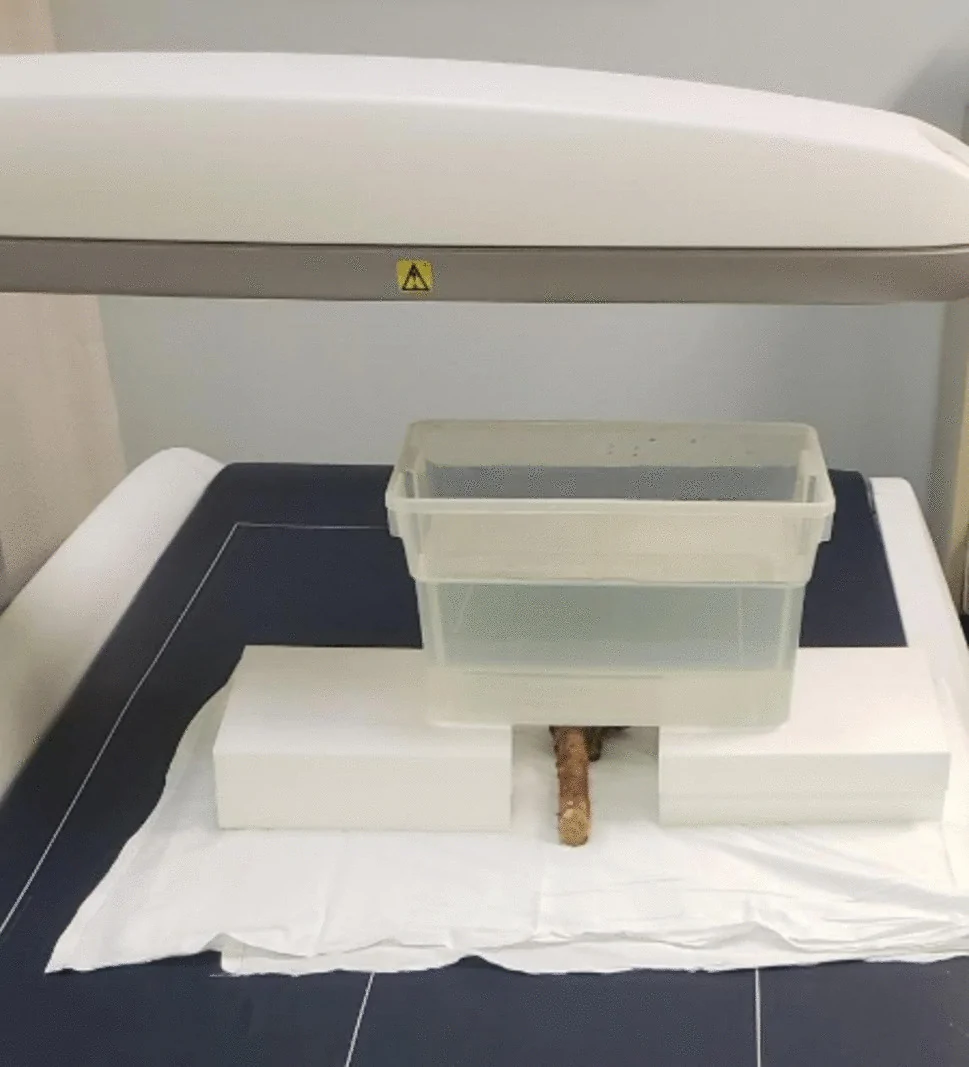
DXA acquisition using water to simulate soft tissue

**Fig. 3:**
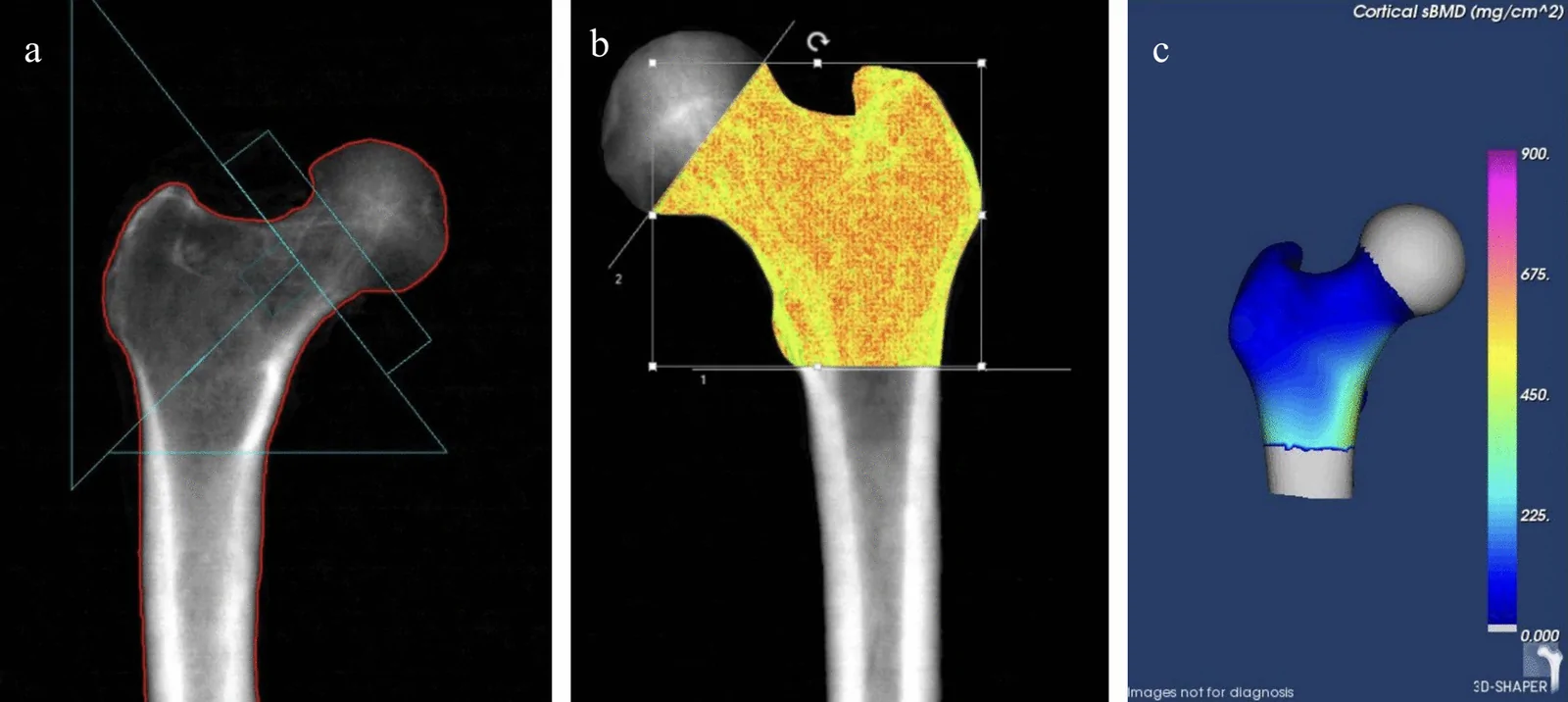
**a** DXA scan analysis using manual adjustments as needed to recreate standard clinical approach to femoral regions of interest (ROIs). Femoral neck ROI shown by rectangle and proximal femur ROI shown by rectangle and triangles combined. **b** TBS Ortho analysis showing a manually drawn ROI to recreate the same proximal femur ROI used for DXA. **c** 3D-Shaper modeling and volumetric measurements using manual adjustments to correctly map the femur and recreate both the proximal femur and femoral neck ROIs used for DXA

After consulting the manufacturer to assist with method development, DXA scans were performed with a Lunar iDXA scanner (General Electric, Madison, WI) using standard hip software (enCORE v18.0). Each femur was internally rotated 15–25° to mimic standard clinical DXA acquisition. A soft tissue simulation was achieved by suspending 15 cm of water above the femur in a plastic container using Styrofoam blocks (Fig. 2). We used standard auto analysis with manual modification to match standard clinical femoral neck and proximal femur analyses (Fig. 3a).

TBS Ortho software (Medimaps, Geneva, Switzerland) was used to generate TBS from exported DXA images using TRIP software version 1.0.1.23. Due to the inability to transfer DXA ROIs directly, we manually recreated the proximal femur ROI within the TBS software (Fig. 3b). We were unable to create an independent femoral neck ROI.

Finally, 3D-Shaper® software v2.12.1 (Barcelona, Spain) generated volumetric bone mineral density (vBMD) data and estimated cortical and trabecular bone mass from DXA images [24]. As the software required pelvic landmarks, manual adjustments were made by the developers to allow correct femoral mapping (Fig. 3c). We collected both femoral neck and proximal femur data.

### Screw placement

**Fig. 4:**
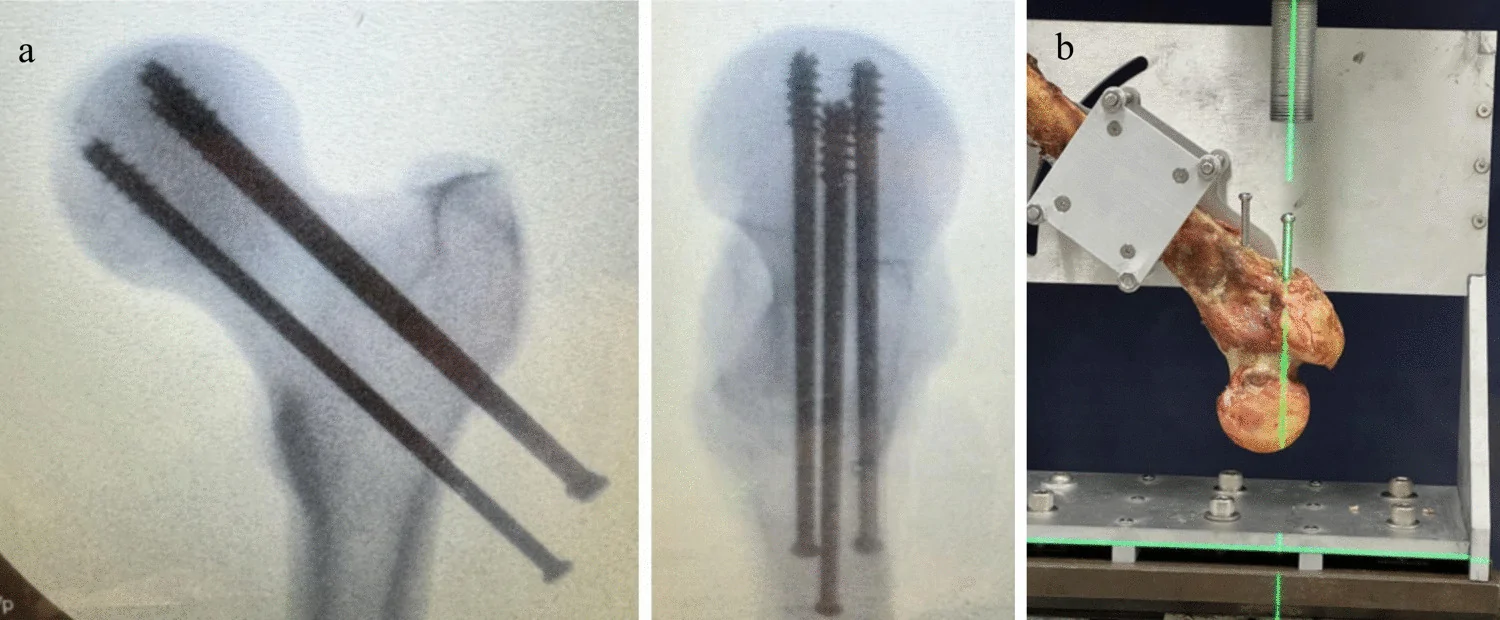
**a** Proper screw placement was confirmed using fluoroscopic imaging prior to biomechanical testing (screws were intentionally longer than necessary to facilitate mechanical testing). **b** Screws were aligned for testing using a laser level and an adjustable fixture. A custom fixture was used to interface with the screw head

Within each femur, we placed three 6.5 mm partially threaded cannulated screws (Depuy Synthes, Raynham, MA, USA) under fluoroscopic guidance (Fig. 4a). To minimize variability, the screws were placed by one author (RJB) in a standard inverted triangle formation for all specimens (Fig. 4a). We used fluoroscopy to confirm optimal depth and trajectory of the screws. The peak insertional torque was measured using a digital torque screwdriver (VPD-P, VANPO; reported range = 0.3–6.0 Nm; maximum errors < 2% of the actual value throughout the measurement range). The peak torque of each screw was measured three times and averaged. Then, the average peak torque of each of the three screws was averaged to get one value per femur. We were unable to register a reading for eight of the sixty screws, likely because the insertional torque was below the minimum detectable threshold of our screwdriver. These screws were excluded from the average torque calculations but were still subjected to pullout testing. We only used the readings available to calculate an average insertional torque (Supplemental Table 1).

### Biomechanical testing

**Fig. 5:**
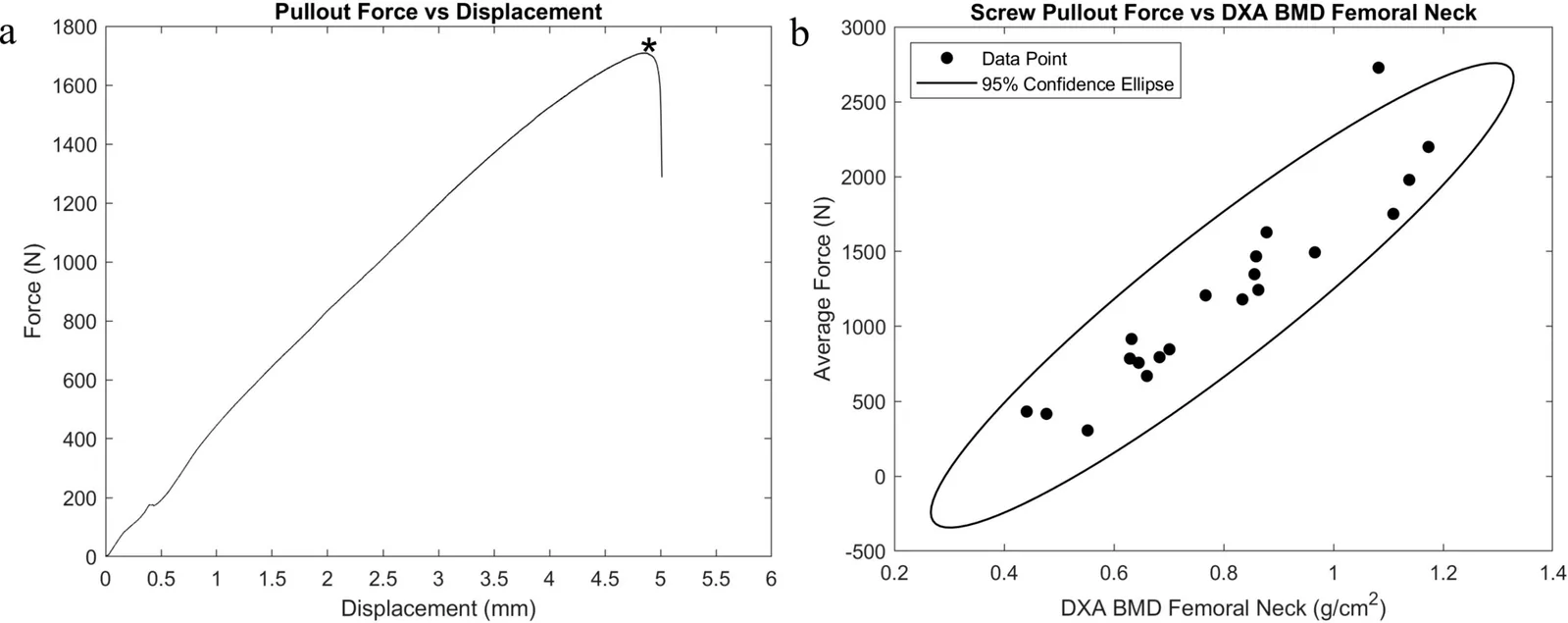
**a** Representative plot of the axial force and screw displacement during the rate-controlled screw pullout tests. The peak force (*) represents the point of screw failure. **b** Representative plot of DXA BMD femoral neck and peak pullout force with a 95% Prediction ellipse (*r* = 0.95, *p* < 0.001)

We rigidly fixed the femurs into a custom stabilizing clamp fixture using two V-clamps around the femoral shaft. The fixture also allowed in-plane rotation to orient each screw to a vertical position. A laser level was used to ensure each screw was coaxial with the MTS machine (858 Bionix, Load Cell 662.20D-05, rated max load = 25 kN ± 0.3%; MTS Systems) (Fig. 4b). After positioning, we used a custom fixture to interface with the screw head (Fig. 4b). Within each femur, screws were pulled out at a rate of 5 mm/s until a force reduction of at least 20% of the maximum force was observed, indicating screw failure (Fig. 5a). The three screws in each femur were pulled out in a random order to minimize any loosening that might occur for subsequent screws (RAND Function, Excel, Microsoft). We extracted the peak force reached before failure for all sixty screws.

### Statistical analysis/study size

Statistical analysis was completed using SAS (version 9.4). We used non-parametric Spearman correlation to determine the strength of relationship between peak pullout force and each assessment of bone quality (i.e., DXA, TBS Ortho, 3D-Shaper, qCT, and insertional torque). Using the bone quality assessments as predictors and a random intercept for each cadaver, we created univariate linear mixed-effects models to determine the amount of variance in peak pullout strength. Spearman correlation coefficients and their 95% confidence intervals (CI) were reported. Mixed-model pseudo-*R*^2^ values were also reported. In all instances, a *p*-value less than 0.05 was considered statistically significant. The sample size included 10 paired proximal femurs (20 total specimens) which was determined based on a previous study that used the distal portion of our femurs. With this sample size and a power of 80%, our study was able to detect a correlation coefficient of at least 0.44 as statistically significant.

## Results

DXA BMD femoral neck and 3D-Shaper vBMD femoral neck had the highest Spearman correlation coefficients with peak pullout force (*r* = 0.95). In addition, DXA BMD proximal femur (*r* = 0.92), qCT femoral head (*r* = 0.89), 3D-Shaper vBMD proximal femur (*r* = 0.88), TBS Ortho proximal femur (*r* = 0.85), and qCT femoral neck (*r* = 0.82) all had statistically significant correlations with peak pullout force (Table 1). Figure 5b shows a representative plot of DXA BMD femoral neck and peak pullout force with a 95% prediction ellipse. This plot displays a strong positive correlation through the tight clustering of data points along a positive trend line with few outliers. Furthermore, we found that 3D-Shaper vBMD femoral neck explained 94.2% of the variance in peak pullout force using univariate linear mixed-effects modeling (*R*^2^ = 0.94). This was the highest *R*^2^ value of any of the predictors; however, the other imaging modalities were also found to explain high levels of variance within the peak pullout force: DXA BMD femoral neck (*R*^2^ = 0.93), qCT femoral head (*R*^2^ = 0.92), DXA BMD proximal femur and qCT femoral neck (*R*^2^ = 0.91), 3D-Shaper vBMD proximal femur (*R*^2^ = 0.91), and TBS Ortho proximal femur (*R*^2^ = 0.91) (Table 1).

**Table 1:** Spearman correlation coefficients and mixed-model pseudo-*R*^2^ values

Bone quality assessment	Spearman correlation coefficient (*r*) [95% CI]^a^	Mixed-model pseudo-*R*^2a^
DXA BMD femoral neck	0.95 [0.82, 0.99]	0.93
DXA BMD proximal femur	0.92 [0.73, 0.98]	0.91
3D-Shaper vBMD femoral neck	0.95 [0.82, 0.99]	0.94
3D-Shaper vBMD proximal femur	0.88 [0.61, 0.97]	0.91
TBS Ortho proximal femur	0.85 [0.53, 0.96]	0.91
qCT femoral head	0.89 [0.64, 0.97]	0.92
qCT femoral neck	0.82 [0.46, 0.95]	0.91
Insertional torque	0.86 [0.56, 0.96]	0.89

^a^All *p*-values were < 0.001 after adjustments for multiple comparisons

Insertional torque was found to have a strong correlation with peak screw pullout force with a Spearman correlation coefficient of 0.86 (Table 1). Additionally, we found that insertional torque explained 89.2% of the variance in peak pullout force (*R*^2^ = 0.89) (Table 1).

## Discussion

Femoral neck fractures are common injuries, especially seen in geriatric patients as a result of low energy falls. These fractures carry high rates of morbidity and mortality; therefore, surgical treatment is crucial to restore function and decrease the risk of poor outcomes [25]. However, multiple complications, such as screw cutout, may occur after femoral neck fracture fixation which may necessitate a return to the OR [26–29]. Our study’s clinical relevance is specifically in patients who have non-displaced or minimally displaced (valgus-impacted) femoral neck fractures but who also have very poor bone quality that might put them at increased risk for failure of screw fixation. In these cases, a surgeon might opt to perform a hemiarthroplasty instead of performing fracture fixation. Thus, it is critical to have validated preoperative assessments of bone quality to predict the risk of implant failure during surgical decision making. Although there are multiple approaches available for the assessment of bone quality, including TBS and 3D-Shaper, the first key finding of our study was that DXA imaging localized to the femoral neck performed as well as these newer imaging modalities when predicting screw pullout strength. Still, we confirmed our hypothesis that TBS Ortho is a strong predictor of screw pullout strength. The second key finding of our study was that insertional torque was a strong predictor of screw pullout strength.

Related to our first key finding, all of the imaging modalities in this study were strong predictors of femoral neck screw pullout strength. Furthermore, those that were specific to the subregion of the femoral neck (i.e., DXA BMD femoral neck, 3D-Shaper vBMD femoral neck) had higher correlation coefficients than those that imaged the broader proximal femur region. However, despite these novel measures that aim to provide more detailed information than standard DXA, our results indicate that standard DXA BMD, localized to the femoral neck, performed as well as the novel alternatives when predicting screw pullout strength. This is inconsistent with a recent study by Bernatz et al. in the distal femur which found TBS Ortho to be a stronger predictor of screw pullout strength than DXA [21]. Interestingly, the Bernatz et al. study did not find that DXA or qCT were correlated to screw pullout strength, whereas our study found that they had a strong correlation. A possible explanation for this difference is the fact that the proximal and distal femurs differ in their composition and distribution of cortical and cancellous bone. Furthermore, there are no standardized or automated approaches to evaluate BMD at the distal femur, which reduces our ability to directly compare to the Bernatz study. An additional study from Eysel et al. found that both qCT and DXA had a strong correlation to screw pullout strength in human vertebral bodies, but that qCT slightly outperformed DXA [30]. In contrast, our study found that DXA performed as well as qCT with both imaging modalities having strong correlations with screw pullout strength [30]. This inconsistency is not surprising as spinal degenerative disease is very common, especially in patients undergoing spine surgery, and is known to artificially elevate spine BMD [31]. Few other studies have investigated how well TBS and vBMD predict screw pullout strength. However, Iki et al. found that vBMD outperformed DXA when predicting fracture risk in the femoral neck of osteoporotic patients [14]. This is in contrast to our results which showed that vBMD and DXA performed the same when predicting screw pullout strength. One possible explanation for this difference is that their study focused on a population of patients with osteoporosis, whereas our cadaver femurs did not have a known history of any conditions that might alter bone density. Additionally, although both studies focused on the proximal femur, the specific ROIs may have varied slightly. A future direction for research will be to define values at which point weakness arises for each of these imaging modalities.

Related to our second key finding, we found that insertional torque was a strong predictor of screw pullout strength in our cadaveric proximal femur model. This agrees with a study from Ab-Lazid et al. which also found that applied insertional torque was a strong predictor of the proximal femur screw pullout strength; however, in contrast to our findings, they found that insertional torque outperformed qCT and DXA [10]. Another study from Zdeblick et al. found that an insertional torque less than 4.0 inch-pounds led to early pedicle screw pullout in the lumbar spine of cadaveric specimens and also outperformed BMD [32]. However, similar to our study, Reitman et al. found that BMD outperformed insertional torque when predicting screw pullout strength in the cervical spine [33]. Together these findings highlight the possible utility of insertional torque in clinical scenarios where imaging such as DXA or qCT are not available preoperatively. Insertional torque has been obtained in the OR via an instrumented torque screwdriver and shown to provide a strong, real-time prediction of bone quality [18, 20].

### Limitations

Six limitations should be considered when interpreting the results of our study. First, it is well known that screws can fail in various ways including pullout/push through, shear, and cutout [3]. However, only screw pullout was represented in this study. Initially, attempts were made to model screw push-through with cyclical loading of the screws to simulate walking. However, the cannulated screws could not withstand the necessary forces without bending. This limitation has minimal impact on the interpretation of our data given that our main objective was to see which imaging modality best correlated with any validated mechanical measure of bone quality. In our study, we chose to use screw pullout because it is a widely used mechanical assessment of bone quality [22, 23]. A second limitation of our study was that a standardized approach to cadaveric DXA measurement does not currently exist in the literature. This limitation also resulted in a need for manual modification of TBS and 3D-Shaper analyses, but it was minimized by using a surrogate to simulate soft tissue and by verifying that the results of our DXA imaging were within the range of what would be expected from in-vivo results. A third limitation was that although the cadaver femurs in this study represented a wide range of ages (50 s-90 s), they did not include any femurs from cadavers younger than 50. Thus, the data may not be applicable to younger adults or pediatric patients. This has minimal impact on the interpretation of our results given that most femoral neck fractures are seen in patients who are 50 or older [34]. The fourth limitation of our study was that we did not calculate the stiffness of the interface between the bone and the screws. To calculate this, we would have needed to use optical tracking to quantify displacement, but we did not have this data available to us. A fifth limitation of our study was that for some of the screws, there was not a high enough purchase in the bone to register on the torque meter. This happened in eight out of the sixty screws; however, we were still able to get at least one reading for every femur. This may have caused an increase in the average torque for those femurs given that we excluded those screws from the average. While we found torque to be strongly correlated with screw pullout, future studies with more sensitive torque meters may be warranted. A sixth limitation is that our study did not delineate cutoff points at which weakness might arise for each respective imaging method. However, this is an area of ongoing research at our institution.

### Conclusion

This is the first study to our knowledge to correlate DXA, TBS Ortho, 3D-Shaper, and qCT with screw pullout strength. We found that DXA BMD of the hip performed as well as these novel imaging modalities. This further emphasizes that regional assessments of BMD are important predictors of hardware failure following fixation of femoral neck fractures. Furthermore, our finding that DXA performed as well as these newer imaging modalities is clinically significant as DXA is already widely available and financially accessible in many clinical settings. One difficulty with DXA is that it is not easily obtainable in the setting of an acute trauma. Therefore, CT may be more useful to traumatologists in predicting when surgical fixation might fail. This is an area of ongoing research at our institution. Additionally, insertional torque served as a strong, real-time assessment of bone quality and predictor of screw pullout strength. These findings may aid in surgical decision-making when treating femoral neck fractures. Future studies are needed to clinically validate these findings in the proximal femur, and to explore these imaging modalities in other commonly fractured anatomic locations.

## Supplementary Information

Below is the link to the electronic supplementary material.

ESM 1 (DOCX 20.6 KB)

## Data Availability

The data that support the findings of this study are available upon request to the corresponding author.
