# Supplementary material for: Predicting femoral neck screw pullout strength using DXA and novel assessments of bone microarchitecture
Source: Arch Osteoporos. 2026 Aug 21;21(1):125. doi: 10.1007/s11657-026-01763-0 (PMC13498487; doi:10.1007/s11657-026-01763-0)
Supplement: Supplementary file 1 [file 11657_2026_1763_MOESM1_ESM.docx]

Article Title: Predicting Femoral Neck Screw Pullout Strength using DXA and Novel Assessments of Bone Microarchitecture

Journal name: Osteoporosis International

Authors: Jarod Moyer, BS^1^ (jrmoyer2@wisc.edu); Richard Behlmer, MD^1^ (rbehlmer@wisc.edu); Diane Krueger, BS^2^ (dckruege@wisc.edu); Gretta Borchardt, BS^2^ (gborchardt@wisc.edu); Neil Binkley, MD^2^ (nbinkley@wisc.edu); Herman Feller, MS^1^ (Feller@ortho.wisc.edu); Sam Mosiman, MS^1^ (mosiman@ortho.wisc.edu); Paul Whiting, MD^1^ (whiting@ortho.wisc.edu); Josh Roth, PhD^1^ ([jdroth2@wisc.edu](mailto:jdroth2@wisc.edu))

*Corresponding Author

Jarod Moyer, BS

University of Wisconsin School of Medicine and Public Health

Department of Orthopedics & Rehabilitation

1685 Highland Ave, 6^th^ Floor

Madison, W1 53705

Phone: 608-263-4069 Fax: 608-263-3430

Email: jrmoyer2@wisc.edu

**Supplemental Table 1:** Peak Screw Pullout Strength and Associated Insertional Torque (red boxes represent screws that did not register on torque-meter)

| Specimen | Side | Screw | Peak Force (N) | Torque (Nm) |
| --- | --- | --- | --- | --- |
| CA15032468 | L | Screw1 | 319.99 | 0.21 |
| CA15032468 | L | Screw2 | 619.51 |  |
| CA15032468 | L | Screw3 | 308.78 |  |
| CA15032468R | R | Screw1 | 249.83 | 0.22 |
| CA15032468R | R | Screw2 | 639.88 | 0.34 |
| CA15032468R | R | Screw3 | 404.86 |  |
| MD14022455L | L | Screw1 | 505.91 |  |
| MD14022455L | L | Screw2 | 828.79 | 0.28 |
| MD14022455L | L | Screw3 | 1206.51 | 0.25 |
| MD14022455R | R | Screw1 | 573.95 | 0.21 |
| MD14022455R | R | Screw2 | 479.81 | 0.24 |
| MD14022455R | R | Screw3 | 953.85 | 0.34 |
| MD14072357L | L | Screw1 | 463.14 |  |
| MD14072357L | L | Screw2 | 917.91 | 0.9 |
| MD14072357L | L | Screw3 | 975.14 | 0.69 |
| MD14072357R | R | Screw1 | 277.79 |  |
| MD14072357R | R | Screw2 | 1416.39 | 0.61 |
| MD14072357R | R | Screw3 | 1054.06 | 0.35 |
| MD14080103L | L | Screw1 | 1749.92 | 0.73 |
| MD14080103L | L | Screw2 | 2935.83 | 2.11 |
| MD14080103L | L | Screw3 | 3497.03 | 3.58 |
| MD14080103R | R | Screw1 | 995.18 | 1.65 |
| MD14080103R | R | Screw2 | 1946.95 | 1.76 |
| MD14080103R | R | Screw3 | 2993.72 | 3.68 |
| MD15031743 | L | Screw1 | 938.3 | 0.29 |
| MD15031743 | L | Screw2 | 1397.13 | 0.49 |
| MD15031743 | L | Screw3 | 1710.48 | 0.77 |
| MD15031743R | R | Screw1 | 1008.14 | 0.37 |
| MD15031743R | R | Screw2 | 1636.8 | 0.94 |
| MD15031743R | R | Screw3 | 2240.18 | 0.78 |
| MD15081843 | L | Screw1 | 777.49 | 1.25 |
| MD15081843 | L | Screw2 | 1885.49 | 2.2 |
| MD15081843 | L | Screw3 | 1820.19 | 1.67 |
| MD15081843R | R | Screw1 | 221.58 | 0.24 |
| MD15081843R | R | Screw2 | 1915.56 | 1.4 |
| MD15081843R | R | Screw3 | 1404.34 | 0.71 |
| MD16031457L | L | Screw1 | 259.41 | 0.2 |
| MD16031457L | L | Screw2 | 319.21 | 0.21 |
| MD16031457L | L | Screw3 | 337.27 |  |
| MD16031457R | R | Screw1 | 477.71 | 0.21 |
| MD16031457R | R | Screw2 | 928.6 | 0.57 |
| MD16031457R | R | Screw3 | 865.81 | 0.43 |
| NJ16102588L | L | Screw1 | 507.36 | 0.24 |
| NJ16102588L | L | Screw2 | 2013.16 | 0.83 |
| NJ16102588L | L | Screw3 | 1211.26 | 0.81 |
| NJ16102588R | R | Screw1 | 683.18 | 0.55 |
| NJ16102588R | R | Screw2 | 1968.51 | 1.41 |
| NJ16102588R | R | Screw3 | 1751.33 | 1.01 |
| TX16091660 | L | Screw1 | 1975.76 | 0.77 |
| TX16091660 | L | Screw2 | 1940.07 | 0.51 |
| TX16091660 | L | Screw3 | 2679.83 | 2.04 |
| TX16091660R | R | Screw1 | 1328.21 | 0.83 |
| TX16091660R | R | Screw2 | 1603.14 | 0.35 |
| TX16091660R | R | Screw3 | 2323.65 | 0.81 |
| VA18052177L | L | Screw1 | 664.15 | 0.31 |
| VA18052177L | L | Screw2 | 1042.63 | 0.36 |
| VA18052177L | L | Screw3 | 676.79 | 0.6 |
| VA18052177R | R | Screw1 | 432.45 |  |
| VA18052177R | R | Screw2 | 1720.65 | 0.87 |
| VA18052177R | R | Screw3 | 1468.6 | 0.88 |
